# How can we encourage engagement in physical activity among older adults in Chinese diasporas? Mixed methods evidence synthesis using the COM-B model

**DOI:** 10.1186/s11556-025-00388-5

**Published:** 2025-11-14

**Authors:** Yang Yang, Lishan Huang, Lily Mott, Kimberly Lazo Green, Nan Zhang, Lisa Mcgarrigle, Chris Todd

**Affiliations:** 1 University of Manchester, Manchester, United Kingdom; 2 Manchester Academic Health Science Centre, Manchester, United Kingdom

**Keywords:** Physical activity, Barrier, Facilitator, Chinese diasporas, Older adults, Systematic review

## Abstract

**Background:**

Globally, 31% of adults are physically inactive, with higher rates in older adults. This situation is even more pronounced among older adults from ethnic minority backgrounds, including those from the Chinese diaspora—a rapidly growing but often overlooked population.

**Objective:**

To explore the barriers and facilitators to engaging in physical activity (PA) among older adults from the Chinese diaspora.

**Methods:**

A mixed-methods systematic review was conducted following Joanna Briggs Institute methodology guidelines. We searched seven databases: Embase, MEDLINE, PsycINFO, CINAHL Plus, Sociological Abstracts, Cochrane Central Register of Controlled Trials, and the Chinese Biomedical Database in September 2024. We included qualitative and quantitative studies related to views and experiences regarding participation in PA of adults aged 50 years or older, from the Chinese diaspora. A thematic synthesis approach was used to summarise the findings. Risk of bias was assessed using MMAT, and confidence in the evidence using GRADE-CERQual. The protocol was registered on PROSPERO (CRD42023392522).

**Results:**

Ten qualitative studies, four quantitative cross-sectional surveys, and one mixed-methods study were included. Twenty-three factors were identified; over half had high certainty. Barriers and facilitators to PA among older Chinese adults aligned with the COM-B model: capability (e.g., health, skills, knowledge, language), opportunity (e.g., PA programme, environment, resources, social support, time), and motivation (e.g., beliefs, concerns, family harmony, enjoyment, habit, reinforcement). Unique factors included language and cultural barriers, family obligations as barriers, and family harmony as a facilitator.

**Conclusion:**

The findings of this review support the development of tailored PA promotion interventions for older adults from the Chinese diaspora by enhancing their capability, opportunity, and motivation. Interventions should incorporate culturally sensitive elements, such as promoting PA benefits through traditional Chinese philosophy, offering traditional exercise programmes, developing Chinese exercise groups, and emphasising family harmony to boost motivation.

**Supplementary Information:**

The online version contains supplementary material available at 10.1186/s11556-025-00388-5.

## Introduction

Population ageing and international migration are two major social transformations observed during the twentieth and twenty-first centuries. The global population of older adults (aged 60 years and over) reached one billion in 2019 and is projected to rise to 2.1 billion by 2050 according to the World Health Organization (WHO) [60]. International migration tends to have the effect of slightly lowering the mean age of a host country as migrants tend to be in younger and working-age groups [47]. However, it is imperative to recognise that once-young immigrants are themselves ageing while continuing to contribute to their host countries [45]. The estimated number of international migrants has more than doubled in the past five decades, reaching around 281 million (3.6% of the global population) in 2020 and approximately 12% of international migrants were 65 years of age or older [24]. The health and well-being of older immigrants have become a significant focus of research and policy analysis, including those who migrated at a younger age and grew old in the destination country, and those who migrated later in life to join their migrant families [8].

Immigrants are generally healthier than native-born residents upon arrival, a phenomenon known as the ‘healthy immigrant effect,’ which is often attributed to the selection of healthier individuals into migration [36]. However, this advantage tends to diminish over time as immigrants experience acculturative stress, face demanding work responsibilities, and encounter structural barriers that hinder the maintenance of healthy lifestyle behaviours, such as engaging in PA [1]. Research shows regular PA plays a vital role in preventing or managing various age-related health conditions [15]. Hupin et al. [22] also report that even a low dose of moderate-to-vigorous PA reduces mortality by 22% in older adults. Regular PA is also associated with a decreased risk of cognitive decline [6] and offers opportunities for socialisation, thereby reducing feelings of loneliness and isolation, which is particularly important for older immigrants [26, 55]. Therefore, promoting PA can be a key strategy to help mitigate health decline and maintain the well-being of older immigrants.

Older adults from the Chinese diaspora represent one of the largest and fastest-growing groups in Western societies [51]. The Chinese diaspora refers to people of Chinese ethnic origin living outside mainland China, Hong Kong, Macau and Taiwan, including first-generation migrants and their descendants [44]. While this review includes all generations, the focus is on older adults; therefore, most study samples consist of first-generation migrants who settled abroad in mid to late adulthood. Similar to older immigrants in general, older adults from the Chinese diaspora often face greater physical and mental health challenges than their native-born counterparts, partly due to the difficulties they encounter in maintaining a healthy lifestyle, including regular participation in PA [65]. A survey of older people in the USA showed that the Chinese ethnic group reported the fewest minutes of weekly vigorous and moderate exercise and total exercise compared with other immigrant groups, and 44% of older Chinese participants reported not attending regular PA [35]. A study of Australian middle-aged and older participants reports that the Chinese ethnic group had a higher prevalence of physical inactivity compared with native Australians [28]. In the UK, among those aged 55–74, the Chinese ethnic group had a lower percentage classified as 'physically active' compared to both the White British population and the national average [18].

The factors influencing PA among older immigrants are multifaceted. Previous reviews among South Asian older immigrants [20], Arab migrants [46], Black and Minority Ethnic groups in the UK [23] and ethnic minorities across Europe [37] similarly point to complex interactions between cultural norms, migration-related challenges, and structural barriers in shaping PA behaviours. Language and cultural factors may affect the willingness of older adults from ethnic minority groups to attend PA [59]. Psychosocial factors, such as knowledge, attitude and health beliefs, can also affect older immigrants’ PA participation [37]. Furthermore, older people from ethnic minority backgrounds usually have fewer choices regarding available PA activities due to cultural and language barriers [12]. However, no systematic review to date has specifically examined older adults from Chinese diaspora communities, despite this group experiencing unique challenges related to socioeconomic factors, language barriers, traditional health beliefs, discrimination, and social isolation [43]. This gap highlights the need for targeted evidence synthesis to better understand the factors influencing PA in this rapidly growing population. Therefore, this review aims to systematically summarise evidence to explore the barriers and facilitators related to PA behaviour among older adults from the Chinese diaspora. The findings from this review can inform researchers, practitioners, and policymakers, providing evidence to support the development of PA promotion initiatives for this population.

## Methods

This systematic review was conducted following the Joanna Briggs Institute (JBI) methodological guidelines for mixed-methods systematic reviews [21]. We report following Preferred Reporting Items for Systematic Reviews and Meta-Analyses (PRISMA) [49]. The review protocol was prospectively registered on PROSPERO (CRD42023392522).

### Literature searches

The original searches were conducted in December 2022 to capture studies reporting the PA behaviour of older adults from the Chinese diaspora. To ensure the review reflects the most recent evidence, an updated search was conducted in September 2024. The six English language databases used are Embase (Ovid), MEDLINE (Ovid), PsycINFO (Ovid), CINAHL Plus (EBSCOhost), Sociological Abstracts (ProQuest) and the Cochrane Central Register of Controlled Trials (CENTRAL). We also searched the Chinese Biomedical Database (CBM) following the recommendation of the Cochrane Handbook for Systematic Reviews of Interventions. Search strategies were developed using a combination of MeSH terms and free-text keywords, incorporating Boolean operators and truncations. Key concepts included “older adults,” “Chinese diaspora/immigrants,” and “physical activity/exercise,” ensuring comprehensive retrieval of relevant literature. The strategies were developed with the assistance of a librarian and are presented in Appendix 1. No restriction was applied regarding the dates of publication. The publications were limited to English and Chinese. Reference lists of eligible studies were screened to identify additional studies.

### Eligibility criteria

We included studies exploring the barriers and facilitators to PA behaviour among community-dwelling older adults in the Chinese diaspora (Table 1). We focused on the Chinese diaspora aged 50 years or above living in the community, including residential and nursing homes. We defined “older adults” as those aged 50 +, consistent with ageing and PA research in immigrant populations [23, 26], where health decline often begins earlier due to migration stress and socioeconomic disadvantages. In addition, 50 + is a widely used cut-off in epidemiological and intervention studies of PA in older adults [38, 58], as intervening in this age group is important for prevention and maintenance of function before disability or frailty becomes established. We accepted any definition or term used in the primary studies, such as migrant, immigrant, or diaspora, as long as they referred to the global community of Chinese ancestry who have emigrated from China and now reside in other countries. There is no limitation on the duration of their residency, however, we excluded studies that focused on Chinese tourists visiting other countries. We included studies that used qualitative, quantitative or mixed-methods.

**Table 1:** Inclusion and exclusion criteria

	Inclusion Criteria	Exclusion Criteria
Population	Older Chinese immigrants/diaspora who were aged 50 years or above and community-dwelling We focused on the Chinese diaspora rather than older adults living in Chinese territories, including mainland China, Hong Kong, Macao, Taiwan, etc	Studies in hospitalised patients and participants with terminal diseases, such as cancer, who may receive specific PA treatment due to their conditions
Context	Studies exploring older Chinese immigrants’ views and experiences related to PA. PA could be one of the multiple topics related to healthy behaviours	Studies that did not relate to PA
Outcomes	Older Chinese immigrants’ views and experiences of barriers and facilitators to engaging in PA	Studies that only describe PA levels without further explanation, or that focused on topics other than PA
Study design	Qualitative study designs (e.g., focus groups or interviews), quantitative designs (e.g., cross-sectional surveys), or mixed-methods studies	Grey literature, commentaries, audits, and review articles

### Study selection and data extraction

Screening of titles/abstracts and full-text was completed independently by two researchers (YY with either LM or LH) using Rayyan [50]. Study details (e.g., study design, data collection method, sample size) and participant information (e.g., age, gender, etc.), guided by PROGRESS-Plus [52] with additional immigrant characteristics, were extracted by two independent researchers (YY with either LM or LH). A pilot phase on a sample of studies was conducted to ensure consistency before full screening and data extraction. Results were compared to ensure agreement, and any disagreements were resolved through discussion. The lead researcher (YY) then extracted data on barriers and facilitators influencing PA from the results and discussion sections.

### Critical appraisal of included studies

Mixed-Methods Appraisal Tool (MMAT) was used to assess the quality of the included studies [19]. MMAT offers a standardised framework for evaluating different study designs, eliminating the need to use separate risk-of-bias assessment tools for each study design. Each study was assessed against five criteria, with each criterion rated as 'Yes', 'No', or 'Cannot tell'. All the assessments were performed by two researchers independently (YY with either LM or LH). Any conflicts were resolved through discussion.

### Confidence in the review findings

As the findings in this review were predominantly supported by qualitative studies and qualitative descriptions of quantitative surveys, we followed the principles of the methodological limitations (quality and risk of bias in the included studies), coherence (consistency and clarity of the findings), adequacy (richness and quantity of the data), and relevance (applicability of the evidence to the review question) from GRADE-CERQual (Confidence in the Evidence from Reviews of Qualitative Research) to critique the confidence of the evidence for each thematic synthesist [39].

All findings started as high confidence and then were downgraded if there were important concerns about any of the CERQual components. Confidence was judged as high, moderate, low or very low. The initial GRADE-CERQual assessment was conducted by the lead researcher (YY) and then discussed with the research team to reach a consensus.

### Synthesis of results

We followed the principle of a convergent integrated approach described in the JBI guideline to inform the evidence synthesis [21]. As per this approach, we used the thematic synthesis method to synthesise data [57]. The initial coding followed an inductive approach, without using any predefined framework, allowing themes to emerge from the data. As the analysis progressed, we found that the factors influencing PA participation among older adults from the Chinese diaspora aligned well with the Capability, Opportunity, Motivation – Behaviour (COM-B) framework [48]. Therefore, an abductive approach was applied, mapping these themes onto the COM-B model to provide a structured, theory-informed interpretation.

Due to resource constraints, the initial coding and analysis were performed by the lead researcher (YY), with final themes discussed and refined within the research team to enhance rigour and validity.

## Results

### Search results

**Fig. 1:**
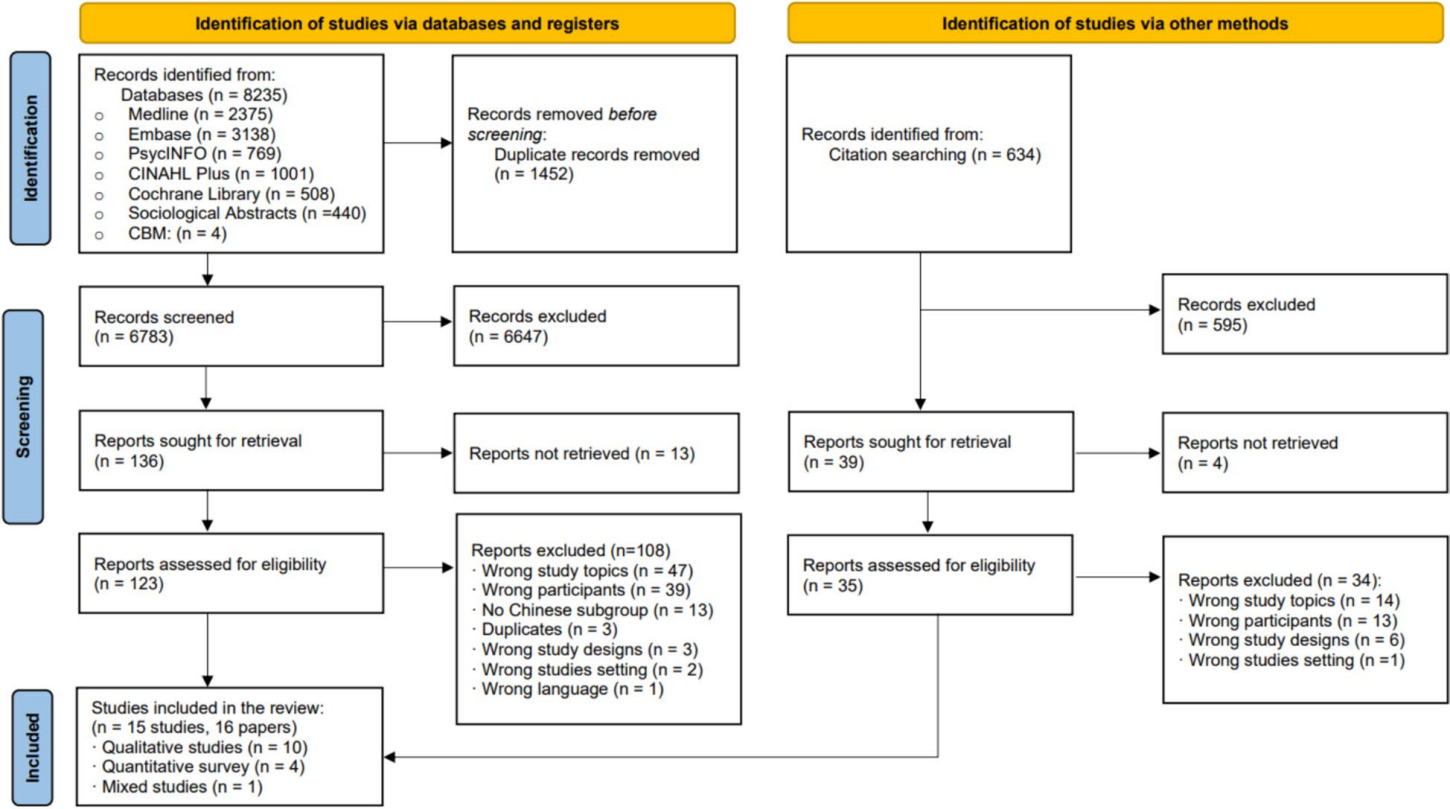
PRISMA flowchart of study inclusion. *From:* Page MJ, McKenzie JE, Bossuyt PM, Boutron I, Hoffmann TC, Mulrow CD, et al. The PRISMA 2020 statement: an updated guideline for reporting systematic reviews. BMJ 2021;372:n71. https://doi.org/10.1136/bmj.n71

The database search yielded 8235 papers, and 6783 papers remained after duplicate removal. Title and abstract screening resulted in 123 potentially eligible papers for full-text review. Full-text screening identified 14 eligible studies (15 papers). One study was identified by checking the references of the included studies (Fig. 1).

### Characteristics of the included studies

The characteristics of the 15 included studies are reported in Appendix 2. The 15 studies (1325 participants) included ten qualitative studies [2, 5, 9, 10, 27, 32–34, 41, 43, 62], four quantitative surveys [17, 40, 42, 53], and one mixed-methods study [30]. The studies were published between 1993 and 2021. Of the 15 studies, nine were conducted in the United States [2, 5, 10, 30, 33, 40–42], three in Australia [9, 32, 34, 62], two in Canada [17, 27], and one in the United Kingdom [43]. The mean age ranged from 63.5 to 75.6 years. There are 680 (51.3%) female participants. The years of living in the host countries varied, ranging from 1 year to more than 50 years.

### Critical appraisal results

The results of critical appraisal using MMAT of the included studies are presented in Table 2. Seven of the ten qualitative studies [5, 10, 27, 32, 41, 43, 62] received “Yes” in all five dimensions. Cerin et al. [9] used a structured qualitative approach and three of the dimensions of this study were assessed as 'cannot tell'. Two quantitative studies reported concerns related to the sampling strategy and sampling representation [17, 42]. The validity and reliability of the measurements were not reported in Li et al. [40] and Parikh et al. [53]. Overall, the studies that used less structured qualitative methodology reported good quality, which was higher than that of other study designs.

**Table 2:** The risk of bias assessment of the included studies by using MMAT (*n* = 15)

Qualitative study	1.1	1.2	1.3	1.4	1.5
Allison and Geiger 1993 [2]	Yes	Yes	Cannot tell	Yes	Yes
Belza et al. 2004 [5]	Yes	Yes	Yes	Yes	Yes
Cerin et al. 2019 [9]	Yes	Cannot tell	Yes	Cannot tell	Cannot tell
Chang et al. 2018 [10]	Yes	Yes	Yes	Yes	Yes
Jette and Vertinsky 2011 [27]	Yes	Yes	Yes	Yes	Yes
Lin et al. 2007 [41]	Yes	Yes	Yes	Yes	Yes
Koo 2011 [32]	Yes	Yes	Yes	Yes	Yes
Liu et al. 2015 [43]	Yes	Yes	Yes	Yes	Yes
Mathews et al. 2010 [33]	Yes	Yes	Cannot tell	Yes	Ye
You et al. 2021 [62]	Yes	Yes	Yes	Yes	Yes
Quantitative survey (*n* = 4)	2.1	2.2	2.3	2.4	2.5
Garcia 2011 [17]	No	No	Cannot tell	Cannot tell	Yes
Li 2015 [40]	Yes	Yes	Cannot tell	Yes	Yes
Liu 2021 [42]	No	No	Yes	Yes	Yes
Parikh 2009 [53]	Yes	yes	Cannot tell	Yes	Yes
Mixed methods study (*n* = 1)	3.1	3.2	3.3	3.4	3.5
Katigbak 2020 [30]	Yes	Yes	Yes	Yes	No

#### Qualitative study

1.1  Is the qualitative approach appropriate to answer the research question?
1.2  Are the qualitative data collection methods adequate to address the research question?
1.3  Are the findings adequately derived from the data?
1.4  Is the interpretation of results sufficiently substantiated by data?
1.5  Is there coherence between qualitative data sources, collection, analysis and interpretation?

#### Quantitative study

2.1  Is the sampling strategy relevant to address the research question?
2.2  Is the sample representative of the target population?
2.3  Are the measurements appropriate?
2.4  Is the risk of nonresponse bias low?
2.5  Is the statistical analysis appropriate to answer the research question?

#### Mixed-method

3.1  Is there an adequate rationale for using a mixed methods design to address the research question?
3.2  Are the different components of the study effectively integrated to answer the research question?
3.3  Are the outputs of the integration of qualitative and quantitative components adequately interpreted?
3.4  Are divergences and inconsistencies between quantitative and qualitative results adequately addressed?
3.5  Do the different components of the study adhere to the quality criteria of each tradition of the methods involved?

### Findings of the thematic synthesis

**Fig. 2:**
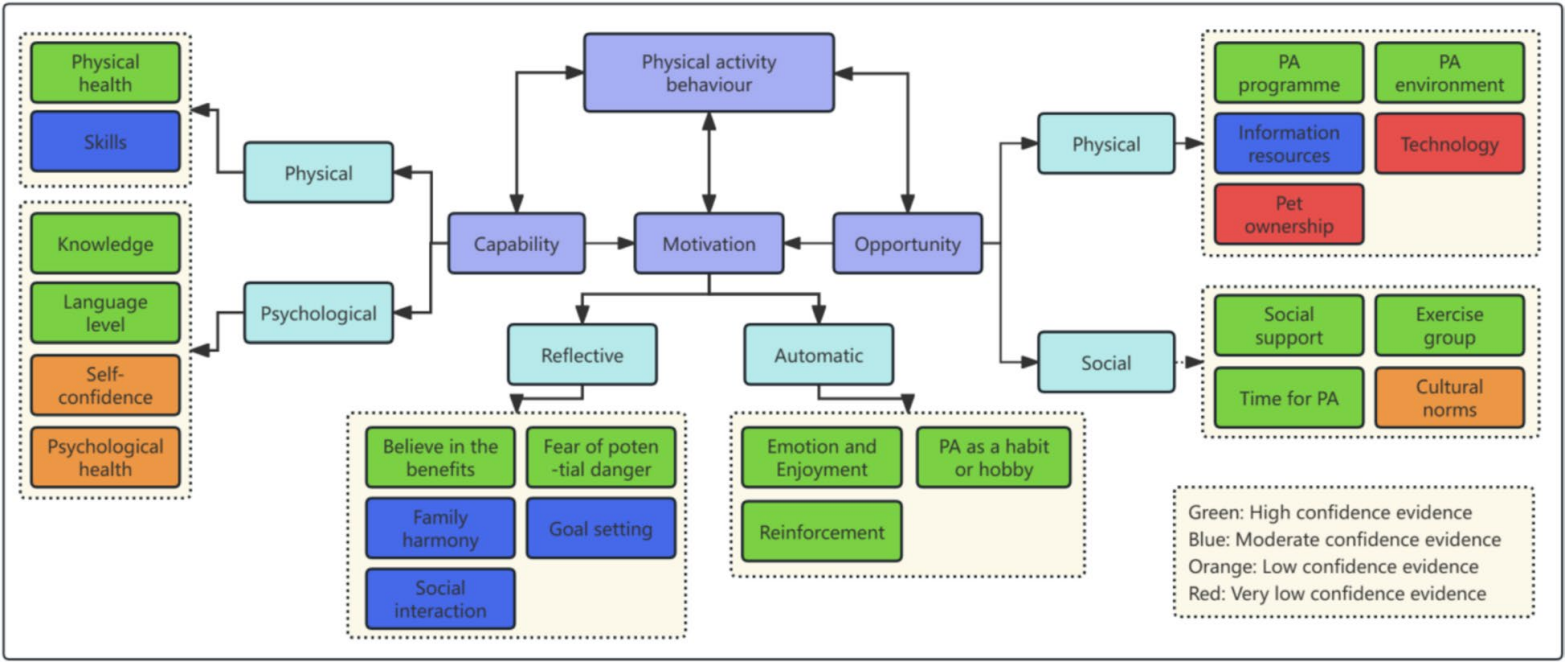
Mapping the factors into the capability, opportunity and motivation

Twenty-three factors influencing PA behaviour among older adults from the Chinese diaspora were mapped to the COM-B framework [48], Fig. 2). Of these, 13 findings were rated as high confidence, five as moderate, three as low, and two as very low confidence (Table 3). High and moderate confidence findings are summarised below:

**Table 3:** The factors influencing physical activity behaviour among older adults from the Chinese diaspora

COM-B theme	Sub-themes	Barriers	Facilitators	Studies contributing to the review finding	Confidence in the evidence	Example of citing of participants or explanation of the author
Capability						
Physical capability	Physical health	- Experiencing physical health problems, such as pain, acute illness, and limited mobility	- Possessing the ability to perform exercise - Offering PA programmes tailored to older adults' health conditions	Ten studies [2, 5, 10, 32, 41, 53,62	High confidence (No or very minor concerns regarding methodological limitations, data coherence, adequacy and relevance)	“Unless I am sick, I will walk every day” [10]. “I often do exercise, so I am in good condition. The other elders can’t crouch down because they are too rigid” [43] “I was a basketball player when younger, but growing old limits my ability. I should be careful to choose suitable types of activities” [41].
	Skills	- Lacking skills in PA	- Possessing PA skills - Providing PA training opportunities	Five studies [2, 27, 30, 34, 62]	Moderate confidence (Downgrade once due to adequacy)	“I exercise two hours every morning, tai chi chuan and kung fu. Very slow motion movements. I have taught tai chi also” [2] “I prefer formal exercise organised by the apartment, because the organiser will invite professionals to instruct us how to exercise” [30]. “I have almost drowned twice at the beach” [34]
Psychological capability	Knowledge	- Lacking knowledge related to PA	- Possessing knowledge about the importance of PA - Having the knowledge to select suitable PA	Five studies [5, 10, 27, 30, 43]	High confidence (No or very minor concerns regarding methodological limitations, data coherence, adequacy and relevance)	“Because when people get to this age, older and older, the natural resistance of the body is weaker and weaker. It is because the physical quality is not as good as it in the youth. Now I keep doing exercise is for enhance my physical quality” [43] “I do some intense exercises on purpose to check chest pain. …… This is very useful for checking the body function …” [43]
	Language level	- Language barrier (e.g., low English fluency)	- Providing PA programmes in Chinese	Six studies [5, 9, 17, 30, 32, 34, 62]	High confidence (No or very minor concerns regarding methodological limitations, data coherence, adequacy and relevance)	“I am very introverted. I don’t know English. I don’t know how to communicate with other people. I’m afraid of saying something wrong. I enjoy staying at home and doing indoor activities” [32] “They don’t know English, so they don’t know there’s this thing (exercise class) so that they can join” [62]. “We don’t speak English and don’t know what is going on outside the apartment” [30].
	Psychological health	- Having difficulty remembering to do exercises	Not Identified	Three studies [5, 32, 53]	Low confidence (Downgrade twice due to adequacy)	“Some, especially women, tended not to practice Tai Chi, despite knowing its benefits, because they thought it was hard to memorise” [32]
	Self-confidence	- Lack of confidence to perform PA	- Confidence to perform PA	Two studies [32, 34, 62]	Low confidence (Downgrade twice due to adequacy)	“I am too old; those people also do not want me there” [32]. “There will be people who aren’t as self-confident who probably don’t go out because they’ll feel embarrassed that they’re walking slowly or can’t walk or have pain and the like” [62].
Opportunity						
Physical opportunity	PA environment	- The home environment does not support PA - Lack of transport - Bad weather (raining, snowing or hot weather)	- Improve neighbourhood safety - Enhance the home environment - Provide affordable transportation options - Offer indoor PA options based on the weather - Establish Chinese community centres - Create a Chinese classical-style PA environment - Ensure access (proximity) to destinations that support PA	Twelve studies [5, 9, 10, 17, 27, 30, 32–34, 40–42, 62]	High confidence (No or very minor concerns regarding methodological limitations, data coherence, adequacy and relevance)	“I guess the weather it prevents you from going out... like going out today when it’s cold and wet. You just keep yourself warm at home, so of course you won’t exercise when you’re home” [62]. The older Chinese …who lived near a park or playground tended to walk more than their Asian counterparts who did not live near a park or playground [40]. “If it’s the right line for wherever the place you want to go to that’s fine, but there are a lot of places you have to change from bus to train, or train to train, or something like that” [62]
	PA programme	- Lack of suitable PA programme - Cost of PA programme	- Easy access to PA programmes - Low-cost PA programmes - Culturally sensitive content tailored to Chinese traditions - Moderate, simple, convenient, and safe PA programmes - Activities integrating physical and mental well-being - Increased community recreational PA opportunities - Recruitment of bilingual/bicultural professionals to address the needs of elderly diaspora populations	Eleven studies [2, 5, 10, 17, 27, 30, 32–34, 41, 43, 62]	High confidence (No or very minor concerns regarding methodological limitations, data coherence, adequacy and relevance)	“Even exercise cannot be overdone. Moderation is the key” [10] “This is (walking) the most common exercise. You don’t need any equipment and you can do it anytime and anywhere. There are no restrictions and it is convenient” [9]. “I come to take part in Tai Chi every week as many other elders and I have done this for nearly 2 months. Tai Chi is very good, good for my health” [43]. “When you write with a Chinese writing brush, your body is working, [and] so are your mind and your brain” [30] “I think there are many sports which are affected by the traditional culture, right? Such like Qigong, Tai Chi Chuan, Tai Chi Sword, calligraphy, embroidery... they are all affected by the traditional culture. These sports emphasise physical and mental self-cultivation” [30]. “If it (Tai Chi) needs to pay, many people will not learn. Older people do not have money” [62] “Walking is the best, you do not need to pay” [62]. “If there are more group activities (provided by this Chinese association) we can walk more … we feel even happier if we go out, we can walk more” [62] “In the gym the exercise is too hard, too heavy for me like walking on the treadmill and all those sort of things you know. I just prefer to take a walk” [62]. “Many old people in their 50 s and 60 s have a lot of energy, but they don’t know where to go and have recreations….The government can organize classes to teach old people how to dance. Through dance classes, old people can be more physically active.” (Mathews 2010)
	Information resource	- Lack of information resources (mainly depend on folk and lay information) - Lack of information resources in the Chinese language - Incorrect information resources may bring harmfulness	- Provding Exercise information in the Chinese language - Providing professional information resources (e.g., guidelines) - Health seminars and education programmes - Television and DVD - Suggestions from friends	Six studies [9, 17, 27, 33, 41, 43]	Moderate confidence (Downgrade once due to methodological limitations)	“Actually, every time I go to see my GP, the conversation with my GP is always very brief … just use some simple words.” [43] “We often talk about how an elder can keep healthy … tips about eating, sleeping and exercise … sometimes, we search this information from internet” [43]
	Use of technology	Not Identified	- Measure PA in an objective way - Provide nice music	Two studies [17, 62]	Very low confidence (Downgraded once due to methodological limitations, and downgraded twice due to adequacy)	
	Pet ownership	- Not Identified	- The presence of pets as companions	Two studies [9, 62]	Very low confidence (Downgraded once due to methodological limitations, and downgraded twice due to adequacy)	“The dog is used to the routine and so she will come to me and say, in her way, come on, time to go, and I dare not go because I care about her too so we’re good for each other”. [62]
Social opportunity	Social support	- Lack of Social Support	- Family support and involvement, - Peer support - Support from health professionals - Support from the community and government	Eleven studies [5, 9, 17, 27, 30, 32–34, 40, 41, 43, 62]	High confidence (No or very minor concerns regarding methodological limitations, data coherence, adequacy and relevance)	“My son told me that the only thing I should do now is taking good care of myself and exercising everyday. He bought a stationary bicycle for me to use at home” [41] “I’d be really pleased if my husband would help me a bit more and I might do more exercise” [62]. “If I have a company, I will have the motivation to play it (Tai Chi) [62]. “My daughter has suggested that I have a morning walk, but I do not want to walk alone because it is very boring” [32] “They should encourage, because they play an important part, the GP, for the older people” [62].
	Time for PA	- Lack of time to perform PA - Social obligations (Family duty; Work duty; Other social obligation (i.e., visit of friends or family)	- Incorporating PA into his daily routine - Having more time after retirement - Reform children activity	Eight studies [5, 9, 10, 17, 27, 30, 34, 62]	High confidence (No or very minor concerns regarding methodological limitations, data coherence, adequacy and relevance)	“The reason we could not do it is because after a long day of hard work, we feel tired. Walking is more like a punishment. In fact, walking is more important than work” [10]. “Grandchildren come to visit once or twice per month. When they invite me, I need to stop other activities and go with them” [30]. “I volunteered as a grandma there for four years. I think this is also a kind of exercise because you need to take care of little kids [30]. “It depends if you can work around it – you can usually work out a schedule of some kind that allows you to get adequate exercise” [62]. “I am a retiree, so I have my set of routine each day. When I get up, I have the time, that certain time that I get up, and I do my exercise, an then I have a shower and whatever” [62]. “For Chinese people in our age, how couldn’t we help? As parents, we are making sacrifice for caring for grandchildren. In doing so we really do not have our own time” [62].
	Ethic-specific PA group	- Lack of Exercise partners and social group - Do not want to have any time restrictions	- Exercise partners - Chinese-speaking social network	Eight studies [9, 10, 27, 30, 32–34, 40, 62]	High confidence (No or very minor concerns regarding methodological limitations, data coherence, adequacy and relevance)	“I have no one who would go out and walk with me. I think it would be really good to have an exercise buddy or group just like this couple who have been exercising together for years” [9].” “I think ethnic-specific senior centres like this are really great to help us old folks” [33] “If there are more group activities (provided by this Chinese association) we can walk more … we feel even happier if we go out, we can walk more” [62]. “It is boring to walk alone” [32] “I don’t want to have any time restrictions,” “I don’t want to socialise with the others in the exercise class” [32].
	Culture norms	- A physical inactivity environment - Culture-related social norms (e.g., not walking with different gender)	- Community or society’s emphasis on PA	Three studies [30, 32, 34, 62]	Low confidence (Downgrade twice due to adequacy)	“In our generation, due to the poor economy, everyone thought about making a living, we had no time to do exercise.. …Now the economy has improved, people can change their lives, such as doing exercise to improve their health” [62]. “I won’t walk with anyone from the opposite sex apart from my wife. It’s related to our Confucian ethical code. It’s not right for me to walk with a woman who is not my wife” [32]. “There’s a culture…You don’t want to trouble other people, to inconvenience people. The Europeans are different [62]. “It seems like we don’t have the same strong desire (to fulfil ourselves) as they [Westerners] do. We just hope to strengthen bodies and develop friendships via exercise” [30]
Motivation						
Reflective	Belief about the benefit of PA	- They believe they are fit and lack the motivation to exercise - Blindly believing in PA can result in a failure to seek help when necessary	- Belief in the benefit of PA on physical health (e.g., keeping and promoting health and body function, preventing disease, controlling weight and enabling longevity - Being aware of the emergence of health problems	Eleven studies [2, 5, 10, 17, 27, 30, 32–34, 41, 43, 62]	High confidence (No or very minor concerns regarding methodological limitations, data coherence, adequacy and relevance)	“The most important reason for doing exercise every day is for health. It is only with health that you can have longevity” [5]. “My mom is over 90 years old, and she exercises every day. She walks four times a day. She used to walk to work from home for over an hour and vice versa” [10]. “Physical activity is very important for maintaining health. No other people but myself can help me keep physically active” [41]. “We need to use all approaches to keep the brain healthy.... This is an issue of all aspects... diet, exercise.” (Mathew et al. 2010) “There is nothing wrong with my heart, so I didn’t take it anymore (calcium channel blocking medication for angina). All because that I did exercise, and my body got better gradually. I do every kind of exercise” [43]. “Every day I insist on doing exercise like this. The regular lifestyle is very good for health” [43]. “Sometimes I was unsteady on my feet and when I wanted to stand up and then I felt a bit wonky… Maybe exercise helps me to handle this problem. Making it better … Now it only depends on doing exercise” [43] “I had a stroke 10 years ago and half of my body could not move. So I started to do exercise, Tai Chi.” [62] “You find that older people who walk or exercise a lot, they live longer lives” [62] “I think it stimulates the brain a bit, yeah. Because especially with the dancing you have to remember the steps...” [62] “I admit that if my health was better, I would not be so enthusiastic about physical activity.” [34] “I know that exercise is good and many people have said so. I don’t do it just because I don’t want to and because I don’t need it. I don’t have any motivation” [34] “diet has a greater effect on health compared to physical activity” [34] “Knowing very well that no one can help me when my legs ache, I have determined not to give up this habit.” [34]
	Being aware of the potential dangers	- Concerns about injuries and harms	- Being aware of the potential dangers can be prevented (choosing a suitable activity)	Six studies [5, 27, 30, 32, 41, 62]	High confidence (No or very minor concerns regarding methodological limitations, data coherence, adequacy and relevance)	“You are worried about falling down thus you don’t want to go out. As long as I see the streets are shiny, I don’t dare to go out. At this age, there is nothing we can do to prevent ourselves from falling down, because our sense of balance is not as good as before when we were young.” [30] “If you choose suitable types of activities for yourself, it would be no harm of exercise.” [41] “I used to ride bike. No matter how far away the destination was, I would ride there. Now I do not have the courage, I am worried I would fall over.” [62]
	Goal setting	Not Identified	-Having clear ultimate goals - Having clear and simple behavioural goals	Five studies [5, 10, 30, 32, 41, 62]	Moderate confidence (Downgrade once due to adequacy)	“I’m not aiming to run 6 kms a day or something, I’m not aiming to do that, I’m just aiming to very, very slowly increase my stamina, I’m already pleased with the amount that I do.” [62]
	Family harmony and unwillingness to be cared for by others	- Unawareness of the benefit of physical activity and believe it is children’s responsibility to take care of them - Unawareness of the necessity for and benefits of self-care	- Demonstrates good health and relieves their children of worry - Desires to be independent and avoid becoming a burden to others. Unwilling to be cared for by others	Four studies [5, 30, 32, 62]	Moderate confidence (Downgrade once due to adequacy)	“When the children call to check up, you can tell them, ‘I am doing good. I go out to walk and do exercises everyday’. Then their families feel more at peace.” [30] “The whole family may have a good feeling towards the fact that the... grandfather or grandmother is engaging in group outdoor activities. This enhances their feeling of happiness and stability. The young [adult children] may feel happier and more secure when out at their workplace.” [30] “I do exercise for the purpose of preventing this (dementia). If I got dementia, my children would live miserable lives” [62] “I do exercise for keeping healthy and not becoming a burden to my children. If I were sick, I would cause trouble to my husband, sons and daughter-in law.” [62]
	The desire for social interaction and to feel less lonely	- Feel social isolation - Prefer easy life	- Opportunity for social engagement - Limited loneliness	Nine studies [2, 9, 10, 17, 27, 30, 32, 41, 62]	Moderate confidence (downgrade once due to methodology concern)	“I retired four years ago. After that, I took part in the dance, Taichi and aerobic exercise at the YMCA every day. Every day I met different people and received different information. It’s just like the old days before retirement, and I never felt lonely. I felt I’m reliving my young life.” [30] Through these exercises, I have made a lot of friends… I have expanded my mind…And talking with friends can keep me from feeling lonely [30] “So, exercise… on the other side, you benefit as well, because you’ve got friends, you’ve got a bigger social circle” [62]
Automatic motivation	Emotion and enjoyment	- Not identified	- Enjoyment - Relief of stress - Benefit mood and spirit	Nine studies [2, 5, 10, 17, 27, 33, 41, 43, 62]	High confidence (No or very minor concerns regarding methodological limitations, data coherence, adequacy and relevance)	“Exercise makes people happy and stop thinking about anything meaningless.” “Walk for a few bus stops, leave my troubles behind.” [5] “you’re walking it takes your concentration away from all the messes going on, and you just feel free to just get on out there and you’re enjoying yourself. you left all that mess behind you, and I think that’s what walking is all about” [10] “When I do tai chi sword, the music and physical posture provided me a feeling of happiness and satisfaction.” [41] It makes me very happy and my mood is very nice [43]
	PA as part of daily routine or hobby	- Lack of interest in PA - Being lazy to attend PA	- Physical activity as a habit - Interests in physical activity	Nine studies [2, 5, 10, 27, 30, 34, 41, 43, 62]	High confidence (No or very minor concerns regarding methodological limitations, data coherence, adequacy and relevance)	“The reason I do not want to walk, as we age, is because I am lazy sometimes. […]. It’s better to make it a ritual or habit. I don’t like to walk but I’m used to it, so I still will go out and walk.” (Change et al., 2018) “Exercise first that’s my priority. Exercise first – everything else will come up to that and then you can plan your time for the other activities” [62] “I remember when I first came to the U.S., I did not know how to drive. As a result, I walked four miles every day to adult school. It would take me over an hour. I was persistent and I did it every day. Now I got lazy and lost my motivation of walking.” [10] “I am lazy, I do not want to do exercise.” [62] “I don’t know how to sort out my worries and make physical activity as part of my life.” [34] “I love doing exercise. I would feel at a loss if I did not perform physical activity.” [34]
	Reinforcement	- Not Identified	- The positive physical experience after PA - The positive psychological experience after PA	Eight studies [2, 5, 10, 27, 34, 41, 43, 62]	High confidence (No or very minor concerns regarding methodological limitations, data coherence, adequacy and relevance)	“Exercise is the activity I do most. ….. My health is good, I feel good. I will always exercise because if I don't, I don't feel good.” [2] “I would not feel well and could not sleep well if I didn’t exercise.” [41] “Every day I do exercise and after it feel very comfortable.” [43]

### Theme 1: capability

#### Physical capability

Eleven studies reported physical capability as a factor, including physical health and skills.

Physical health, including health status, disease, and chronological age, was the most frequently reported barrier across ten studies [2, 5, 9, 10, 17, 32, 34, 41, 43, 53, 62]. Poor physical health, such as pain, limited mobility, and low energy levels, was reported as potentially preventing older adults from starting or maintaining PA.

Skills are reported as another capability-related factor, as evidenced in five studies [2, 27, 30, 34, 62]. Participants with physical skills, such as performing Tai Chi are more likely to engage in PA, while those lacking those skills see this as a barrier. This is also why older adults appear to prefer exercise classes led by a professional, as it provides a chance to learn the necessary skills [30].

#### Psychological capability

Eleven studies identified factors related to psychological capability: knowledge related to PA and language proficiency.

Five studies identified knowledge as a factor related to psychological capability [5, 10, 27, 30, 43]. Participants who understand how PA benefits health appear to be more open to engaging in PA. Knowledge related to PA can also help older adults choose suitable exercises, while a lack of trustworthy knowledge may pose potential dangers. For example, one participant in Liu et al. [43] mentioned engaging in intense exercise to see if it induced chest pain, as a way to check their body’s functioning.

Language level, specifically poor English fluency, was reported as a barrier to attending PA in six studies [5, 9, 17, 30, 32, 34, 62]. It also influences the PA choices of the participants. For example, participants reported avoiding outdoor activities due to language barriers, opting instead to exercise at home [32, 34].

### Theme 2: opportunity

#### Physical opportunity

Fourteen studies reported physical opportunities to influence participants' PA behaviour. These factors include the PA environment, PA programmes and information resources.

The PA environment was consistently identified as being very important regarding physical opportunity by twelve studies [5, 9, 10, 17, 27, 30, 32–34, 40–42, 62]. Factors identified include proximity to activity-friendly destinations, reliable public transport, supportive home environments, safe spaces, and quality facilities. Other environmental factors include weather, for example, rain or cold weather may prevent older adults from going out. In addition, Liu et al. [42] reported that culturally sensitive environments promote outdoor walking among elderly Chinese immigrants in the United States.

The types of PA programmes available is another factor related to physical opportunity, which was reported by eleven studies [2, 5, 10, 17, 27, 30, 32–34, 41, 43, 62]. Preferred characteristics of PA programmes include safety, convenience, affordability, moderate intensity, simplicity, and the integration of mental and physical components. Studies indicate a preference for culturally sensitive activities, such as Tai Chi. Walking was another frequently reported PA behaviour, valued for its simplicity and convenience, and was the focus of three studies [10, 40, 42].

Six studies [9, 17, 27, 33, 41, 43] identified the availability of information resources, particularly those related to PA in the Chinese language, as a valuable facilitator. Participants received PA recommendations or information from health professionals, books, newspapers, television, DVDs or journals. A local Chinese association also can provide health seminars and information on local exercise groups [41].

#### Social opportunity

Social opportunity, arising from social factors, was reported in thirteen studies. Three factors were identified: social support, exercise group or buddy, and time for PA.

Social support emerges as the most common facilitator for PA engagement, which was reported in eleven studies [5, 9, 17, 27, 30, 32–34, 40, 41, 43, 62]. This encompasses various forms of support, such as family support (e.g., providing equipment, transportation, and encouragement), recommendations from healthcare professionals, and peer influence and support. Conversely, a lack of support or negative influences from family and peers can act as barriers to PA participation.

Moreover, as an ethnic minority group, older adults from the Chinese diaspora reported challenges in finding PA buddies who share the same language and culture, while the availability of Chinese activity groups were recognised as a facilitatonullr [9, 10, 27, 30, 32, 34, 40, 33, 62]. Many older Chinese adults find walking alone boring [32], while living near other Chinese elders and experiencing neighbourhood social cohesion are seen as facilitators [9, 40]. However, some individuals may avoid group activities due to a preference for flexible scheduling and a desire to minimise social interaction within an exercise setting [32, 34].

Another factor is having time to engage in PA [5, 9, 10, 17, 27, 30, 34, 62]. Participants were usually occupied with family responsibilities and work commitments during their younger years, with more free time becoming available after retirement. However, many older adults from the Chinese diaspora reported a persistent lack of time due to ongoing social obligations, most notably family duties such as caring for grandchildren.

### Theme 3: motivation

#### Reflective motivation

Eleven studies reported reflective motivation, which refers to the type of motivation that arises from an individual's self-reflection and internal processes. Four findings were identified: beliefs about the benefits of PA, worry about the potential danger, family harmony, desire for social interaction and goal setting.

Beliefs about the benefits of PA on well-being were reported in 11 studies [2, 5, 10, 17, 27, 30, 32–34, 41, 43, 62]. Participants regarded PA as essential for maintaining health and longevity, identifying it as their primary motivator. They also saw PA as beneficial for mental health, reducing depressive symptoms and stimulating cognitive function. Many participants reported initiating PA following health issues, with the belief that exercise could aid in improving their condition. This belief is often shaped by traditional Chinese philosophy, which views PA as a means of maintaining bodily balance. However, some participants lacked motivation, seeing themselves as fit and not needing PA or prioritising factors like diet over PA.

In contrast, worry about injury and falls can reduce motivation [5, 27, 30, 32, 41, 62]. Participants may avoid going out due to concerns about falling. Some participants indicated that selecting suitable types of activities enhances safety.

Family harmony was another motivation. They hold the belief that PA is crucial for maintaining independence, thus avoiding becoming a burden to their children and family [5, 62]. Participants also believe engaging in PA demonstrates good health, relieving their children of worry [30]. However, some study participants are unaware of the need for and benefits of self-care and believe it is their children's responsibility to take care of them, which could reduce their motivation for PA [32].

The desire for social interaction was reported as another motivation [2, 10, 17, 27, 30, 32, 41, 62, 9]. Participants engage in PA to build friendships, enhance social connections, and alleviate loneliness.

Moreover, by setting specific, achievable goals such as increasing daily step counts, individuals can feel a sense of accomplishment and track their progress, further motivating them to maintain their PA routines [5, 10, 30, 32, 62].

#### Automatic motivation

Automatic motivation refers to automatic processes that influence individuals' engagement in PA, was reported in thirteen studies. Three factors were identified: enjoyment, PA as a habit and reinforcement.

Enjoyment and stress relief during and after PA are the most frequently reported automatic motivations [2, 5, 10, 17, 27, 33, 41, 43, 62]. They are more likely to engage in PA automatically if they enjoy activities such as dancing or walking, as these make them feel happy.

When PA becomes a habit and part of daily routine, it will serve as an automatic motivation [2, 5, 10, 27, 30, 34, 41, 43, 62]. In addition, studies indicate that individuals who were physically active in their younger years are more likely to remain physically active in older age [5]. Conversely, a lack of interest was reported as a barrier to incorporating PA into daily routines [10, 34, 62].

Reinforcement, which refers to the positive physical and psychological experiences gained from participating in PA, served as a reward and automatically motivated individuals to continue engaging in it [2, 5, 10, 27, 34, 41, 43, 62]. Participants reported feeling good and experiencing better sleep after exercise but feeling uncomfortable when they skipped it.

## Discussion

This is the first mixed-methods review to explore the barriers and facilitators influencing PA behaviour among older adults from the Chinese diaspora. The findings show that PA behaviour is shaped by three interactive factors: capability, opportunity, and motivation, which align with the COM-B model. We discuss these factors, reflecting the barriers to and facilitators of PA shared with the general ageing population, those related to being part of an ethnic minority group, and the unique factors associated with Chinese culture. We also examine their implications for developing culturally appropriate PA promotion strategies.

Similar to the general ageing population [56], high-confidence evidence supports the important role of physical health in influencing PA behaviour. Poor physical health, such as chronic pain, reduced mobility, or multiple long-term conditions, often limits their physical capability to participate in PA, particularly moderate-intensity activities, and to meet PA guidelines for older adults [25]. Conversely, those with better health status are more likely to initiate and sustain PA, as they experience fewer physical restrictions and perceive greater benefits. This highlights the need for suitable PA programmes, for example, chair-based exercises for individuals with poor health or limited activity history, with a focus on light, regular activity rather than strict adherence to the full WHO recommendation [7].

Building knowledge of the importance of PA and skills is a crucial factor that contributes to developing participants’ psychological capability, which then further increases their motivation, making individuals more likely to engage in PA. Knowledge of PA guidelines is often limited among older adults, especially those from disadvantaged groups [31]. Evidence on the link between PA knowledge and behaviour is mixed: Cheung et al. [11] found no association between guideline awareness and PA behaviour, whereas Fredriksson et al. [16] reported higher activity among those aware of diseases linked to inactivity. Therefore, PA interventions should not only incorporate education to raise awareness, build skills, and emphasise benefits, but also integrate evidence-based strategies to optimise engagement.

Another factor influencing psychological capability is the language barrier, supported by high-confidence evidence and consistent with findings in other ethnic minority groups [23, 37]. As a capability-related factor, the language barrier can further limit opportunities and motivation, as individuals may feel uncomfortable attending local PA programmes or may struggle to understand information provided in the local language. Programmes led by Chinese instructors and delivered in Chinese can create a culturally inclusive environment, addressing language barriers and promoting participation.

This review found high-certainty evidence that physical opportunities strongly influence PA behaviour. As in the general ageing population, factors such as accessible destinations, transport, weather, home safety, and neighbourhood characteristics shape older adults’ engagement in PA [4]. Older Chinese adults tend to prefer moderate-intensity, simple, and slow-paced activities that emphasise safety and convenience, often rooted in traditional philosophy, such as Tai Chi [29]. However, these preferences may not fully meet WHO guidelines, which recommend moderate-to-vigorous activity for achieving optimal health benefits [61].

High-confidence evidence highlights an association between social support and PA among older adults from the Chinese diaspora, reflecting trends observed in the general older population [37]. Family members, in particular, play a crucial role in facilitating PA, as noted by Yuan et al. [63]. Additionally, moderate-confidence evidence suggests that older adults from the Chinese diaspora may engage in PA for social interaction. Exercise buddies who share the same language and culture, along with Chinese exercise groups, provide both opportunities and motivation for PA participation [59]. Incorporating family-oriented, multi-generational, and culturally familiar group activities, as well as offering bi-cultural options such as classes using Chinese music and establishing buddy systems, may strengthen social support, boost motivation, and promote sustained participation.

Social obligations are a major barrier to PA among older Chinese adults. Rooted in cultural expectations that emphasise family roles, many prioritise childcare and household responsibilities even after retirement [64]. These obligations often limit time for PA, unlike other groups with fewer familial demands. Interventions should therefore engage family members or integrate PA into daily routines to improve participation.

Consistent with previous studies, belief in the health benefits of PA and the desire to maintain health are the most common motivators for engagement among older adults [14, 54]. The onset of health issues often prompts older individuals to recognise the importance of PA for health improvement, serving as a common catalyst for initiating engagement in PA [27]. Furthermore, older adults often engage in PA to maintain their health and avoid becoming a burden to their children and family. This motivation is deeply rooted in the traditional concept of filial piety, or “xiao,” in Chinese culture, where adult children are expected to care for their ageing parents. However, this expectation has shifted in recent years, with many older adults expressing a desire for greater independence and self-reliance [13]. For these individuals, PA is seen not only as a way to preserve functional ability but also as a means to maintain autonomy and reduce the caregiving burden on their families. These motivations ought to be incorporated into the design of interventions.

Experiencing physical and psychological benefits from PA can foster automatic motivation and help older adults establish routines that reinforce enjoyment and sustained engagement [3]. Interventions should emphasise early benefits, address safety concerns, and increase motivation and gradually integrate PA into daily routines to support habit development and long-term adherence.

### Limitations and strengths

This mixed-method systematic review has a number of strengths. Firstly, the review followed a mixed methods approach to integrating all relevant studies on this topic, providing a broader understanding of the barriers and facilitators for older adults from the Chinese diaspora to engage in PA. Secondly, a rigorous review and synthesis process was employed, including following the CERQual principle to assess confidence in the findings. Finally, this review adopted COM-B as a theoretical framework to underpin the evidence synthesis of barriers and facilitators. This framework enhances the conceptual clarity and organisation of the findings, allowing for a systematic analysis of the factors influencing PA participation.

However, this review has some limitations. A key limitation is that coding was conducted by a single researcher, which may have introduced bias despite team discussions to mitigate this risk. In addition, only English and Chinese publications were included, and grey literature was excluded, which may have led to publication bias and limited cultural insights. Future studies are encouraged to incorporate additional languages and grey literature to enhance cultural insights and provide more comprehensive evidence.

### Implications

This review identifies that factors influencing PA behaviour align with the COM-B model, which forms the core of the Behaviour Change Wheel (BCW). This suggests the BCW could be used to develop PA promotion interventions for older adults from the Chinese diaspora. Culturally appropriate PA interventions should be safe, accessible, and low-cost, address language and cultural barriers, foster social support, emphasise family benefits, help overcome time constraints, and encourage gradual activity increases informed by WHO recommendations. When applying these findings, it is important to note that over half of the evidence was rated as high confidence, exemplified by social support, providing a strong foundation for culturally tailored interventions and policies. Moderate-confidence findings, such as family harmony and goal setting, warrant further validation, whereas low and very-low-confidence findings, including cultural norms and technology, should be interpreted with caution and prioritised for future research.

## Conclusion

The facilitators and barriers influencing PA behaviour among older adults from the Chinese diaspora align closely with the COM-B model. These factors include general elements affecting older adults, such as health conditions, knowledge, motivation, and access to suitable programmes, as well as culturally specific needs, including language barriers, cultural preferences, and family obligations. This review provides a comprehensive evidence base to guide the development of culturally appropriate interventions to promote PA in this population.

## Supplementary Information

Supplementary Material 1

Supplementary Material 2

## Data Availability

No datasets were generated or analysed during the current study.

## Abbreviations

CBM: Chinese biomedical database
COM-B framework: Capability, opportunity and motivation-behaviour framework
BCW: Behaviour change wheel
GRADE-CERQual: Confidence in the evidence from reviews of qualitative research
PA: Physical activity
WHO: World health organization
