# Supplementary material for: How can we encourage engagement in physical activity among older adults in Chinese diasporas? Mixed methods evidence synthesis using the COM-B model
Source: Eur Rev Aging Phys Act. 2025 Nov 14;22:22. doi: 10.1186/s11556-025-00388-5 (PMC12619512; doi:10.1186/s11556-025-00388-5)
Supplement: Supplementary file 1 — Supplementary Material 1 [file 11556_2025_388_MOESM1_ESM.docx]

# Appendices

## Appendix 1 Search strategies for MEDLINE <1946 to January 2024>

1 Aged/ 3402848

2 middle aged/ 4741582

3 (old* or elder* or senior* or aged or ag?ing or geriatric* or pensioner* or retire* or "late life" or late-life or "later life" or later-life or "later lives" or later-lives or longevity).mp. [mp=title, book title, abstract, original title, name of substance word, subject heading word, floating sub-heading word, keyword heading word, organism supplementary concept word, protocol supplementary concept word, rare disease supplementary concept word, unique identifier, synonyms] 6837893

4 ("middle aged" or "middle life" or "midlife" or "mid-life" or "midlives" or "mid-lives").mp. [mp=title, book title, abstract, original title, name of substance word, subject heading word, floating sub-heading word, keyword heading word, organism supplementary concept word, protocol supplementary concept word, rare disease supplementary concept word, unique identifier, synonyms] 4752960

5 ("over 50" or "over fifty" or "over 55" or "over fifty-five*" or "over 60" or "over sixty" or "over 65" or "over sixty five*" or "70" or "seventy").mp. [mp=title, book title, abstract, original title, name of substance word, subject heading word, floating sub-heading word, keyword heading word, organism supplementary concept word, protocol supplementary concept word, rare disease supplementary concept word, unique identifier, synonyms] 703875

6 1 or 2 or 3 or 4 or 5 7216864

7 Minority Groups/ 17087

8 Minority Health/ 891

9 Population Groups/ 5462

10 "Ethnic and Racial Minorities"/ 603

11 "Emigrants and Immigrants"/ 14765

12 "Transients and Migrants"/ 13748

13 "Emigration and Immigration"/ 26156

14 Ethnicity/ 70891

15 Cultural Diversity/ 12788

16 Human Migration/ 1581

17 Refugees/ 12615

18 ((cultural* or language*) and (adapt* or accomodat* or approp* or target* or tailor*)).mp. [mp=title, book title, abstract, original title, name of substance word, subject heading word, floating sub-heading word, keyword heading word, organism supplementary concept word, protocol supplementary concept word, rare disease supplementary concept word, unique identifier, synonyms] 66054

19 (refugee* or diaspora* or immigrant* or transient* or migrant* or asylum or refugee* or (asyl* adj1 seek*) or foreign* or ethnic* or minorit* or racial* or (multi adj cultural*) or multicultural* or (newly adj arrived) or ((family or families) adj2 reuni*) or resettlement or (cultural adj1 (diversit* or pluralism))).mp. [mp=title, book title, abstract, original title, name of substance word, subject heading word, floating sub-heading word, keyword heading word, organism supplementary concept word, protocol supplementary concept word, rare disease supplementary concept word, unique identifier, synonyms] 788870

20 7 or 8 or 9 or 10 or 11 or 12 or 13 or 14 or 15 or 16 or 17 or 18 or 19 858573

21 Chinese/ 73367

22 China/ 237428

23 (China or Chinese or Hong kong or "Tai Wan" or Macao or Mandarin or Cantonese).mp. [mp=title, book title, abstract, original title, name of substance word, subject heading word, floating sub-heading word, keyword heading word, organism supplementary concept word, protocol supplementary concept word, rare disease supplementary concept word, unique identifier, synonyms] 517217

24 21 or 22 or 23 553895

25 Exercise/ 136921

26 Motor Activity/ 100296

27 Exercise Therapy/ 47671

28 "Physical Education and Training"/ 14045

29 Physical Fitness/ 29349

30 Life Style/ 62807

31 Leisure Activities/ 9683

32 Sedentary Behavior/ 13146

33 Dancing/ 3393

34 Sports/ 33797

35 Tai Ji/ 1394

36 Walking/ 39930

37 Yoga/ 3659

38 (physical adj (fitness or education or training)).mp. [mp=title, book title, abstract, original title, name of substance word, subject heading word, floating sub-heading word, keyword heading word, organism supplementary concept word, protocol supplementary concept word, rare disease supplementary concept word, unique identifier, synonyms] 50702

39 ("physical activit*" or "physically active").mp. [mp=title, book title, abstract, original title, name of substance word, subject heading word, floating sub-heading word, keyword heading word, organism supplementary concept word, protocol supplementary concept word, rare disease supplementary concept word, unique identifier, synonyms] 127178

40 ("physical inactivit*" or "physically inactive").mp. [mp=title, book title, abstract, original title, name of substance word, subject heading word, floating sub-heading word, keyword heading word, organism supplementary concept word, protocol supplementary concept word, rare disease supplementary concept word, unique identifier, synonyms] 9906

41 (exercise* or sport* or strength or balance or resistance or MVPA).mp. [mp=title, book title, abstract, original title, name of substance word, subject heading word, floating sub-heading word, keyword heading word, organism supplementary concept word, protocol supplementary concept word, rare disease supplementary concept word, unique identifier, synonyms] 1835670

42 ((sedentary adj (lifestyle or behavio?r)) or sedentariness).mp. [mp=title, book title, abstract, original title, name of substance word, subject heading word, floating sub-heading word, keyword heading word, organism supplementary concept word, protocol supplementary concept word, rare disease supplementary concept word, unique identifier, synonyms] 18502

43 (aerobic* or swim* or aqua* or jog* or run* or cycl* or walk* or danc* or fitness or yoga or sport* or Tai Chi).mp. [mp=title, book title, abstract, original title, name of substance word, subject heading word, floating sub-heading word, keyword heading word, organism supplementary concept word, protocol supplementary concept word, rare disease supplementary concept word, unique identifier, synonyms] 2045261

44 25 or 26 or 27 or 28 or 29 or 30 or 31 or 32 or 33 or 34 or 35 or 36 or 37 or 38 or 39 or 40 or 41 or 42 or 43 3683596

45 6 and 20 and 24 and 44 2189
