# Supplementary material for: How can we encourage engagement in physical activity among older adults in Chinese diasporas? Mixed methods evidence synthesis using the COM-B model
Source: Eur Rev Aging Phys Act. 2025 Nov 14;22:22. doi: 10.1186/s11556-025-00388-5 (PMC12619512; doi:10.1186/s11556-025-00388-5)
Supplement: Supplementary file 2 — Supplementary Material 2 [file 11556_2025_388_MOESM2_ESM.docx]

Appendix 2 Characteristics of Included Studies (n=15)

| Study ID  Country | Study Design Study aims | Participants characteristics  Age Mean (SD, range) Gender N (%) female Clinical condition Place of Residence  Length since immigrant  Place of birth  First language  Fluence of English | Data collection method Language for data collection  Data analysis method | Main findings |
| --- | --- | --- | --- | --- |
| Qualitative studies (n=10) | | | | |
| Allison and Geiger 1993 United States | Study design: Qualitative study The study aims: to identify and analyse the patterns and nature of leisure activity among elderly Chinese-American people | Sample size: 25 Participants: Individuals who were over age 60, were born in south-eastern Asia, and spoke at least one Chinese dialect. Age: 64 to 85 Female: n=15 (60%) Clinical condition: NR  Place of residence: Community (a southwestern metropolitan city) Length since immigrant: 40%< 20 years, and 60% > 20 years. Place of birth: All of the respondents were from mainland China; First language: 76% spoke Cantonese, 20% spoke Mandarin, and 4% spoke Toishanese; respondents (56%) spoke English as their second language Fluency in English: Slightly more than half of the respondents (56%) spoke English as their second language, with varying degrees of proficiency. | Data collection: Open-ended personal interviews  Language: Data were collected in Chinese or English (the number of interviews in each language was not reported)  Data analysis: Data were analysed for recurring patterns and themes and interpreted within the cultural context of the respondents' daily lives. | Tai chi, dancing, walking, house working and gardening were the reported physical activity  The perceived benefits of physical activity included: staying in shape and controlling weight, feeling young, enjoying, benefiting physical health, spending time alone and to maintaining their traditions and heritage. Preferring Chinese traditionally activities. |
| Belza et al. (2004) United States | Study design: Qualitative study Study aims: to examine barriers and facilitators to physical activity and exercise among underserved, ethnically diverse older adults | Sample size:9 Participants:  Age: 69.4 (SD 3.71)  Female: n=6 (66.7%) Clinical condition: NR  Place of residence: Community  Age when immigrant:54.8 [51-67] Length since immigrant (year): 14.6 (4.69), [6-22] First language: Cantonese Fluency in English: NR | Data collection: Focus groups Language: Cantonese  Data analysis: all team members code one transcript and develop a framework and then code the remaining transcripts. | Common themes:  PA as health promotion The complex role of Chronic Conditions Family as encouragement Environmental barriers (weather; neighbourhood safety; fear of crime; programme costs; and inadequate availability, frequency, and reliability of affordable transportation) The importance of a daily activity routine Dressing appropriately so can walk in the rain  Choose PA depending on the weather  Social obligations interfere with an exercise routine.  Suggested features of PA programmes (e.g., fostering relationships among participants; providing culture-specific exercise; offering programmes at residential sites; partnering with and offering classes prior to or after social service programmes; educating families about the importance of physical activity for older adults and ways they could help; offering low- or no-cost classes; and involving older adults in programme development) |
| Cerin et al. (2019)  Australia | Study design: Qualitative study Study aim: to examine built and social environmental facilitators of and barriers to regular engagement in physical activity, eating a healthy diet and regular contact with other people. | Sample size: 37 Participants: older adults from the Chinese diaspora Age: 60-64:n=11 (29.7%); 65-69: n=6 (16.2%); 70-74: n=6 (16.2%); 75-79: n=11 (29.7%); 80: n=3 (8,1%) Female: n=23 (62.1%) Clinical condition: NR  Place of residence: Community (urban Melbourne) Length since immigrant: <5 Years 20 (54.1%); 4-15: 6 (16.2%); 15-24 years: 5 (13.5%); 25+years: 6 (16.2%) Place of birth: Mainland China: 32 Hong Kong, Taiwan, Indonesia, or Vietnam: 5  First language: Mandarin only: 20 (54.1%); Cantonese only: 7 (18.9); Others: 10 (27.0%) Fluency in English: NR | Data collection: Nominal Group Technique (NGT, a qualitative, structured, brainstorming technique).  Language: Mandarin or Cantonese  Data analysis: The responses from NGT groups were aggregated, an inductive thematic analysis was used to identify higher order theme. | Facilitators: Proximity to destinations; Easy access to destinations for physical activity; Access to social groups; Community health even information in Chinese; Home environment, Social support; Enjoying household physical activity; Opportunities to facilitate integration in the community; Quality public transport; Pet ownership; Safety from traffic; Caring responsibilities; Living near other Chinese elders  Barriers: Poor public transport; Language barriers ; Lack of destinations supporting physical activity ; Limited social group/activities; Health-related and socio-economic factors; Lack of information on community activities home environment not conducive to physical activity; Lack of social support; Lack of public housing supporting independence |
| Chang et al. (2018) United States | Study design: Qualitative study Study aim: to understand older people’s beliefs of stoke and PA to inform the develop culturally tailored stroke prevention walking intervention | Sample size: 34  Participants:>60 years, from Chinese ethics minority group  Age: 69 (SD 5) Female: n=23(67.6%) Clinical condition: hypertension Place of residence: Community  First language: Mandarin (not exclude Cantonese) Fluency in English: 7/34 are very comfortable with English | Data collection: Focus Group Language: Mandarin  Data analysis: thematic analysis, | Perceived benefit of Walking: Physical health and Safe and convenience, chance to socialisation, improve neighbourhood  cohesion Perceived barriers: physical health (e.g., Acute illness), lack of motivation, competing priorities (e.g., caring for grandchildren), Lack of exercise partner |
| Jette and Vertinsky (2011)  Canada | Study design: Qualitative study Study aim: understand how the exercise beliefs and practices of older Chinese women diaspora | Sample size: 15 Participants: Chinese-origin women aged 65 and over, and who were actively participating in exercise classes or sporting/fitness activities Age: 70 [66-80] Female: n=15 (100%) Clinical condition: NR  Place of residence: Community  Length of diaspora and fluence of English: NR | Data collection: In-depth, semi-structured interviews Language: Two interviews were conducted in English and thirteen in Cantonese.  Data analysis: thematic analysis | Participants reported that they usually re/turned to Chinese exercise in an attempt to regain health after a stressful or busy lifestyle led to what they perceived as ‘health fatigue.  Suggestions from the professors and friends could be the enables to attend PA.  The perceived benefit of physical activity helps them to maintain the behaviour. |
| Koo (2011/2012)  Australia | Study design: Qualitative study Study aim: to explores how older Hong Kong Chinese Australians perceive ageing and to what extent this perception affects their participation in physical activities  Theory: the theory of planned behaviour | Sample size: 22 Participants: Older Cantonese-speaking Chinese people who had migrated to Australia from Hong Kong Age: 75.5 (60 to 91 years) Female: n=10 (45.5%) Clinical condition: NR  Place of residence: NR  Length since immigrant: 2-21 years Place of birth: Mainland or Hong Kang First language: Cantonese Fluency in English: over half (55%) had poor English skills | Data collection: Semi-structured in-depth interviews Language: Cantonese  Data analysis: content analysis | Attitudes toward physical activity: A perception of susceptibility to illness (Doing exercise because of their age; Wanting to lose weight or keep fit). Capacity to care for themselves: (Trying to avoid injury. Unwilling to be cared for by others). Unawareness of the necessity for and benefits of self-care (Don’t think too much about Children’s responsibility to take care of them) Social support: family (Wanting to exercise with family. Their families were too busy. It is boring to walk alone); Friends (Good friends who regularly do exercise would influence those who did not. Afraid of saying something wrong or being rejected). Personality (Being introverted. Like quiet. Like doing exercise alone. Having a sense of inferiority. Not wanting to have any time restriction). Barriers to participation in PA: deterioration of physical condition or injury, deterioration of mental condition, cultural issues and lack of transportation. |
| Lin et al. (2007) United States | Study design: Qualitative study The study aims: to compare the beliefs about physical activity held by Chinese immigrant older adults in Seattle and Chinese elderly in Taipei.  Theory: theory of planned behaviour (TPB) | Sample size: 10 Participants: Chinese, aged 65 years or older Age: 75-79 years: n=6 (60%) 80-87 years: n= 4 (40%) Female: n=8 (80%) Clinical condition: NR  Place of residence: Community | Data collection: Focus group Language: Mandarin or Taiwanese  Data analysis: Deductive content analysis | Activities: walking, tai chi, gymnastics, shopping, light housework, and light gardening. Physical activity can benefit physical health, relaxation stress, limited loneliness. Family member (spouses and children) and friends can influence physical activity behaviour.  Information resources and Chinese community service centre (providing health seminars and education programmes) are also important for them to attending physical activity.  Education teachers as important referents.  Internal motivation, interest in PA and health condition are the factors that make physical activity easy or difficult. |
| Liu et al. (2015)  UK | Study design: Qualitative study (A Grounded Theory approach) The study aims: to explore behaviours and attitudes towards exercise among older Chinese adults in the UK.  Theory used: NR | Sample size: 33 Participants: older Chinese adults in the UK  Age: 71 [60–84] Female: n=23 (69.7%) Clinical condition: NR  Place of residence: NR  Years living in the UK (mean duration) 1.5–54 years (25years) | Data collection: in-depth semi-structured face-to-face interviews.  Language: Mandarin or Cantonese  Data analysis: substantive coding followed by theoretical coding (constant comparison method) | The frequently mention physical activity: Tai Chi.  Believe that exercise is key to maintaining physical and psychological well-being. The ability to engage in exercise can serve as an indicator of one's health condition.  Older adults often prefer gentle, generally non-vigorous, and slow exercises, such as Tai Chi and walking. They engage in physical activity on a daily basis, typically in the early morning.  The lack of appropriate information from professionals can lead them to rely on folk beliefs or information from peers.  Exercise can also serve as a barrier to seeking help when they believe it can be a panacea for all health problems. |
| Mathews (2010)  United States | Study design: Qualitative study  The study aims: to identify perceived PA enablers and barriers among  a ethnically diverse group of older adults.  Theory used: NR | Sample size: 36 Participants: Older Chinese adults living in the community  Age: 69.0 (8.3)  Female: n=25 (69.4%) Clinical condition: NR  Place of residence: Community | Data collection method: Focus group.  Language: English, Cantonese or Mandarin  Data analysis: the constant-comparison method was used for the discovery of similarities and differences in the data. | There are no barriers identified related to PA among the Chinese American subgroup.  Four enablers were reported: the expectation of positive outcomes; feeling better (i.e., feeling younger, having more energy, and becoming relaxed); social support; having access to PA Facilities and Programmes |
| You et al. (2021)  Australia | Study design: phenomenological  Approach (Individual and group interviews)  Study aims: to examine the perceived benefits of and barriers and enablers to PA from the perspectives of older Caucasian and Chinese adults living in Australia  Theory used: Social-Ecological Model (SEM) | Sample size: 47  Participants: Community-dwelling, older (≥60 years Chinese adults living in Australia Age: 74.0 (8.5) Female: n=33 (70.2%) Clinical condition: NR  Place of residence: Community  Years living in the US: 25.7 (13.7);  Prefer languages: Mandarin or Cantonese as first language | Data collection method: Semi-structured (mix of individual or focus group)  Language: English, Mandarin or  Cantonese  Data analysis: thematic analysis | The Perceived Benefits of PA including physical and functional benefit, mental heath and brain health.  The barriers and enablers match with Social-Ecological Mode:  The Individual level barriers: health issues, lack of time, lack of motivation, older age, concerns about injuries and harms, lack of interest, feeling tired, individual ability, lack of knowledge and lack of confidence. The individual related enablers included maintaining or improving health, self-motivation, enjoyment, maintaining a habit, having a fulfilling life, having more time after retirement, goal setting, having a pet, use of technology, self-discipline,  Individual and interpersonal related barriers: language and culture-related barriers, lack of family or peer support. Individual and interpersonal related enablers: peer influence or support, social aspects of exercise, unwilling to become a burden on children, people’s compliment,  Community or organisation related barriers: cost concerns (affordability), bad weather, distant exercise location, anti-exercise environment. Environment related enablers: Pro-exercise culture and PA opportunities |
| Survey studies and mixed methods studies (n=5) | | | | |
| Katigbak et al. (2020)  United States | Study design: a mixed-methods study Study aim: to better understand older Chinese Americans’ perceptions of physical activity | Sample size: 59 Participants: Mandarin- or Cantonese-speaking adults, >55 years old, AGE: 75.6 (SD 8.77) Female: n=38( 64.4%) Clinical condition: NR  Place of residence: Community  Length since immigrant:15.8 (±10.14) Place of birth: Mainland China 55 (94.8); Hong Kong 1 (1.7), Singapore 1 (1.7); Taiwan 1 (1.7) Preferred Language: Chinese (Cantonese or Mandarin): n=58 (98.3%); English: 1 (1.7%) | Data collection: Survey and focus groups Language: The questionnaires were translated into Chinese The focus groups were conducted in Mandarin or Cantonese.  Data analysis: Survey data was analysed by descriptive statistics approach; Qualitative data were independently coded by two coders, who then discussed the codes and grouped commonly occurring ones into higher-order themes. | Physical activities benefit the body and mind.  Traditional Chinese culture influences perceptions and preferences for physical activity.  Physical activity presents opportunities for social engagement; and physical activity facilitates family harmony. Design recommendations include encouraging mind and body approaches, incorporating culturally specific practices, highlighting opportunities for social engagement, and emphasizing the potential for improved harmony. |
| Garcia and Da (2011)  Canada | Study design: Quantitative survey  Study aim: to explore the dietary and physical activity profiles of older Chinese diaspora and some sociocultural factors influencing their adaptations | Sample size: 31 Participants: older Chinese diaspora aged ≥55 years who had resided in Canada for ≤13 years Age: male: mean age, 71 years female: mean age, 69 years Gender: n=20 (64.5%) Clinical condition: NR  Place of residence: Community  Length since immigrant:15.8 (±10.14) Place of birth: Mainland China 55 (94.8); Hong Kong 1 (1.7) Singapore 1 (1.7); Taiwan 1 (1.7) First language: Mandarin-speaking Fluency in English: male: Fair: 36%; Poor 64% Female: Fair: 35%; Poor: 65% | Data collection: Semi-quantitative instruments and qualitative data (i.e. free-text comments of the participants) Language: The questionnaire was translated into Chinese (Mandarin) language.  Data analysis: descriptive analysis approach and qualitative data were included to explain some of the findings. | The mentioned PA types included: walking, gardening and housework, Tai Chi and other forms of Chinese body movements, playing ping pong, climbing stairs, exercising by following a TV programme and swimming. Reasons for doing PA: to improve health, to reduce risk of health problems, for enjoyment/fun, to improve physical condition or to get stronger, to socialise with friends, to lose weight, and to feel better or release stress.  Barriers: not enough time, no place to exercise, interferes with family/work and social activities, no programmes available in the Chinese language or no transportation, poor physical condition or not enough energy to exercise, and bad weather/snow. |
| Li et al. (2015)  United States | Study design: Cross-sectional survey  The study aim: to examine the associations between neighbourhood factors and walking. | Sample size: 355 Participants: older adults aged 55 and above Age: 55-64: 41%; 64-74: 36%; 75+: 22% Female: n=199 (56.1%) Clinical condition: NR  Place of residence: Community | Data collection: Cross-sectional interview survey Language: Mandarin and Cantonese  Data analysis: statistical analysis using Stata. | Social cohesion  Nearby park Safe neighbourhood. |
| Liu et al. (2021)  United States | Study design: Visual preference survey Study aim: to examine the relationship between visual preference and motivation to walk in a particular outdoor space | Sample size: 95 Participants: first-generation Chinese elderly immigrants Age: 60–69: n=4 (4%); 70–79: n=25 (26%); 80–89:n=45 (47%); >=90: n=21 (22%) Female: n=61 (64.2%) Clinical condition: NR  Place of residence: NR Age when immigrant: NR Years living in the United States: 10-20: n=11 (12%); 11–20: n=19 (20%); 21–30: n=29 (31%); 31–40: n=17 (18%); >=41: n=9 (9%) Place of birth: Mainland China: n=73 (77%); Hong Kong n=3 (3%); Taiwan n=2 (2%); Other (e.g., Vietnam, Malaysia, Indonesia) 17 (18%) | Data collection: Visual preference survey  Language: Not reported  Data analysis: Descriptive statistics and factorial experiment design | Participants reported a high preference for Chinese classical walking spaces. |
| Parikh et al. (2009) United States | Study design: cross-sectional survey Study aim: to examine the prevalence and predictors of PA associated with chronic disease prevention.  Theory used: NR | Sample size: 517 Participants: Chinese diaspora aged 55 years and older Age: 63.5 (SD 0.38) Female: n=181 (35.0%) Clinical condition: NR  Place of residence: Community  Years living in the US: 25.9 (0.9);  Prefer languages: English, Mandarin, Cantonese, and Fukinese. | Data collection: Cross-sectional in-person interviews survey  Language: English, Mandarin, Cantonese, and Fukinese  Data analysis: Multivariable logistic regression | Participants with higher education, higher income, longer proportion of their lives in the U.S and better physical health scores were more likely to engage in physical activity. |
